# Usability Assessment of a Cable-Driven Exoskeletal Robot for Hand Rehabilitation

**DOI:** 10.3389/fnbot.2019.00003

**Published:** 2019-02-13

**Authors:** Yu-Lin Tsai, Jian-Jia Huang, Shu-Wei Pu, Hsiang-Peng Chen, Shao-Chih Hsu, Jen-Yuan Chang, Yu-Cheng Pei

**Affiliations:** ^1^Department of Physical Medicine and Rehabilitation, Chang Gung Memorial Hospital at Linkou, Taoyuan, Taiwan; ^2^School of Medicine, Chang Gung University, Taoyuan, Taiwan; ^3^Department of Power Mechanical Engineering, National Tsing Hua University, Hsinchu, Taiwan; ^4^Healthy Aging Research Center, Chang Gung University, Taoyuan, Taiwan; ^5^Center for Vascularized Composite Allotransplantation, Chang Gung Memorial Hospital at Linkou, Taoyuan, Taiwan

**Keywords:** upper distal limb rehabilitation, stroke, rehabilitation device, usability, exoskeleton

## Abstract

**Study design:** Case series.

**Background:** Robot-assisted rehabilitation mediated by exoskeletal devices is a popular topic of research. The biggest difficulty in the development of rehabilitation robots is the consideration of the clinical needs. This study investigated the usability of a novel cable-driven exoskeletal robot specifically designed for hand rehabilitation.

**Methods:** The study consists of three steps, including prototype development, spasticity observation, and usability evaluation. First, we developed the prototype robot *DexoHand* to manipulate the patient's fingers based on the clinical needs and the cable-driven concept established in our previous work. Second, we applied *DexoHand* to patients with different levels of spasticity. Finally, we obtained the system usability scale (SUS) and assessed its usability.

**Results:** Two healthy subjects were recruited in the pre-test, and 18 patients with stroke and four healthy subjects were recruited in the formal test for usability. The total SUS score obtained from the patients and healthy subjects was 94.77 ± 2.98 (*n* = 22), indicating an excellent level of usability. The satisfaction score was 4.74 ± 0.29 (*n* = 22), revealing high satisfaction with *DexoHand*. The tension profile measured by the cables showed the instantaneous force used to manipulate fingers among different muscle tone groups.

**Conclusions:** *DexoHand* meets the clinical needs with excellent usability, satisfaction, and reliable tension force monitoring, yielding a feasible platform for robot-assisted hand rehabilitation.

## Background

Robot-assisted rehabilitation has become increasingly popular in recent decades, and there are more and more therapy concepts that attributed to various novel rehabilitation robots (Shields et al., 1997; DiCicco et al., 2004; Wege and Hommel, 2005; Hasegawa et al., 2008; Chiri et al., 2009; Tadano et al., 2010; Brokaw et al., 2011; In et al., 2011; Kadowaki et al., 2011; Ueki et al., 2012; Villafañe et al., 2017; Cerasa et al., 2018; Jakob et al., 2018; Mazzoleni et al., 2018; Pila et al., 2018). In robot-assisted rehabilitation, patients can receive standardized and repetitive training controlled by robot actuators (Takahashi et al., 2005; Chang and Kim, 2013). Most of these robots are applied to disabled limbs, such as those with motor impairments. To this end, the design of these robots is based on clinical needs, and the usability and satisfaction are the foundation of success before clinical application (Pei et al., 2017).

Stroke is a cerebrovascular disorder that affects the brain (Calautti and Baron, 2003; Kwakkel et al., 2008; Sampaio-Baptista et al., 2018) and the major etiology of neurological disability. The World Health Organization reported that there are about 15 million new stroke cases worldwide each year, and one third of them need long-term rehabilitation (Benjamin et al., 2017; Thrift et al., 2017). Spasticity is a neurological symptom commonly observed in stroke, manifested as an involuntary, velocity-dependent increase in muscle tone, and thus causing resistance to movement (Wu et al., 2007; Lin et al., 2009). Spasticity hinders movement ability, thereby limiting patients' activities of daily living.

A human hand is an extremely dexterous end effector that is necessary for performing daily activities such as grasping, writing, driving, or picking (Burton et al., 2011). However, the study (Feys et al., 1998) found that, in most stroke patients, the recovery of motor function in the upper limb is slower than that in the lower limb. Several rehabilitation techniques are presently adopted for hand rehabilitation, such as task-oriented motor training (Bayona et al., 2005; Fischer et al., 2007), continuous passive movement (CPM) therapy (Hu et al., 2009; Tzemanaki et al., 2011), bimanual therapy (Gordon et al., 2007), and mirror therapy (Yavuzer et al., 2008). Each technique has a number of advocates and has proven effective. However, up to 20–60% of stroke patients still have difficulty using their affected limb after completion of treatment (Kwakkel et al., 1999). To this end, it is necessary to develop rehabilitation devices used in the rehabilitation facilities to realize novel therapeutic strategies for improving upper limb function.

In terms of robot-assisted rehabilitation products for hands, *Hand of Hope* (Rehab-Robotics Company Limited, HK) and *Sinfonia* (Gloreha Inc., Italy) are commercially available on the market (Troncossi et al., 2016). *Hand of Hope* is an exoskeletal hand robot whose movement can be trigged by surface electromyography (Tong et al., 2010; Ho et al., 2011). The mechanical design of the exoskeleton covering the fingers allows patients to manipulate their fingers with considerable spasticity. The robot is supported by a desk due to the heavy weight of the exoskeleton and five actuators. Sinfonia is much more lightweight as the fingers are moved by a glove actuated through five cables (Varalta et al., 2014; Villafañe et al., 2017), and each cable is controlled by an actuator housed in a control box. Sinfonia uses the design of soft glove, and the force that manipulates the finger is applied to the distal phalanx (Bissolotti et al., 2016; Gobbo et al., 2017). The way of the force is applied to the finger, and inadequate structure around the finger make it unable to precisely control each of the finger joint angles, including the metacarpophalangeal (MCP), proximal interphalangeal (PIP), and distal interphalangeal (DIP) joints. Furthermore, the soft glove might make it difficult to manipulate fingers in severe spasticity. Given the dichotomy between these two designs, it is necessary to propose a method that can have the advantages of both designs and avoid the disadvantages. A recent review of robot-assisted hand rehabilitation found a total of eight exoskeleton devices, but none of which is similar to our design (Yue et al., 2017).

According to the study of Lu et al. (2011), some physical therapists considered that clinically usable hand rehabilitation robots should accommodate different hand movements and can be used in seated postures, provide feedback, and restore patients' activities of daily living. We developed an exoskeleton prototype, *DexoHand*, in this study based on the concept of our previous works (Pu et al., 2015, 2016, 2017). It is a cable-driven exoskeletal robot in which the exoskeleton allows patients with spasticity to manipulate their fingers, while the cable driven design keeps the motor away from the exoskeleton to reduce the weight imposed on their hands. Furthermore, the one-to-one correspondence between the motor position and the finger position makes it possible to provide precise joint angle control. Finally, tension sensors are placed on each of the cables that drive the flexion and extension movement so that the force provided by the actuators can be precisely measured (Pu et al., 2015). We apply CPM, a simple and standardized rehabilitation movement, to examine the repeatability of movements and the changes of cable tension across repetitive movements and movement velocities. Stroke is a syndrome with a wide spectrum of clinical presentation and severity. For robot-assisted rehabilitation for the hand, spasticity is hypothesized to impose tremendous difficulties on a variety of aspects of usability. For example, the clenched fist needs to be relieved before application of the robot, making it more difficult when wearing the robot. This kind of problem will make it more time consuming or even induce discomfort as each finger needs to be manually extended to fit the exoskeleton. In this study, we recruited stroke patients with different spasticity levels and assessed the patient's usability and satisfaction for the perceived when receiving the CPM therapy. First, we hypothesized that, by applying a physiologically aspired movement pattern, the device would yield good to excellent usability. Second, we hypothesized that spasticity will affect the usability, with lower scores of usability for patients with stronger spasticity.

## Methods

This study consists of three steps: (1) prototype development; (2) spasticity observation; and (3) usability evaluation. To develop the device, we adopted the design concept from previous study (Pu et al., 2017), and revised prototype according to the clinical needs. We respectively used standard joint angle measurement and the Modified Ashworth Scale (MAS) to obtain the subjects' physical and neurological properties, including range of motion (ROM) and spasticity. Finally, for the usability and satisfaction assessments, we applied *DexoHand* to the subjects' hands and evaluated the tension profile recorded by the cable tension sensors along the movement trajectories. The subjects were evaluated by subjective evaluations (including usability and satisfaction) after application.

### Subjects

This study was approved by the Institutional Review Board of Chang Gung Medical Foundation (No. 105-6337C). Two adult healthy subjects were recruited for the pre-test and the data were not used in the present study. From 1st June 2017 to 31th January 2018, 22 adult subjects were recruited for the formal test, including 4 healthy subjects and 18 patients with stroke (male: female = 15:3, aged 20–75 years; Table 1). All subjects signed the informed consent before participation. In order to reduce cross-subject variance, only supratentorial stroke patients were included. For healthy subjects, the inclusion criterion was healthy adults with no physical disabilities. The inclusion criteria for the stroke patients were: (1) stable medical and neurological conditions, (2) brain lesions were supratentorial, (3) right hemiplegia, (4) the MAS assessment of spasticity in the right hand was 0, 1, 1+, 2, or 3, and (5) normal cognitive and language skills. The exclusion criteria for both groups were: (1) fractures affecting the upper limbs during the first 3 months, or (2) active skin lesions. The subjects were not paid for participation.

**Table 1:** Demographic information from the 22 subjects.

**Subject number**	**Status**	**Age (year)**	**MAS**	**Experience of using rehabilitation robot**
01	Stroke patient	56–60	1	No
02	Stroke patient	51–55	1	No
03	Stroke patient	46–50	1	No
04	Stroke patient	71–75	1	No
05	Stroke patient	56–60	1	No
06	Stroke patient	56–60	1	No
07	Stroke patient	46–50	1	No
08	Stroke patient	66–70	1	No
09	Stroke patient	51–55	1	No
10	Stroke patient	66–70	1+	No
11	Stroke patient	56–60	1+	No
12	Stroke patient	61–65	1+	No
13	Stroke patient	36–40	1+	No
14	Stroke patient	51–55	1+	No
15	Stroke patient	51-55	2	No
16	Stroke patient	41–45	2	No
17	Stroke patient	41–45	2	No
18	Stroke patient	36–40	3	No
19	Healthy subject	26–30	0	No
20	Healthy subject	41–45	0	No
21	Healthy subject	61–65	0	No
22	Healthy subject	26–30	0	No

*MAS, Modified Ashworth Scale*.

The subjects provided basic biographical information before assessment. We then explained the experimental process and demonstrated the operation of the device. The device was operated by researchers for usability assessment. The primary outcome was the system usability scale (SUS) questionnaire and the secondary outcomes were satisfaction questionnaire, ROM, and MAS assessments. During data analysis, patient subjects (*n* = 18) were divided into three groups of MAS _≦1_ (1 ≦ MAS, *n* = 9), MAS_1+_ (MAS = 1+, *n* = 5), and MAS _≧2_ (MAS ≧ 2, *n* = 4) according to spasticity levels.

### Step 1: Device Development

**Figure 1:**
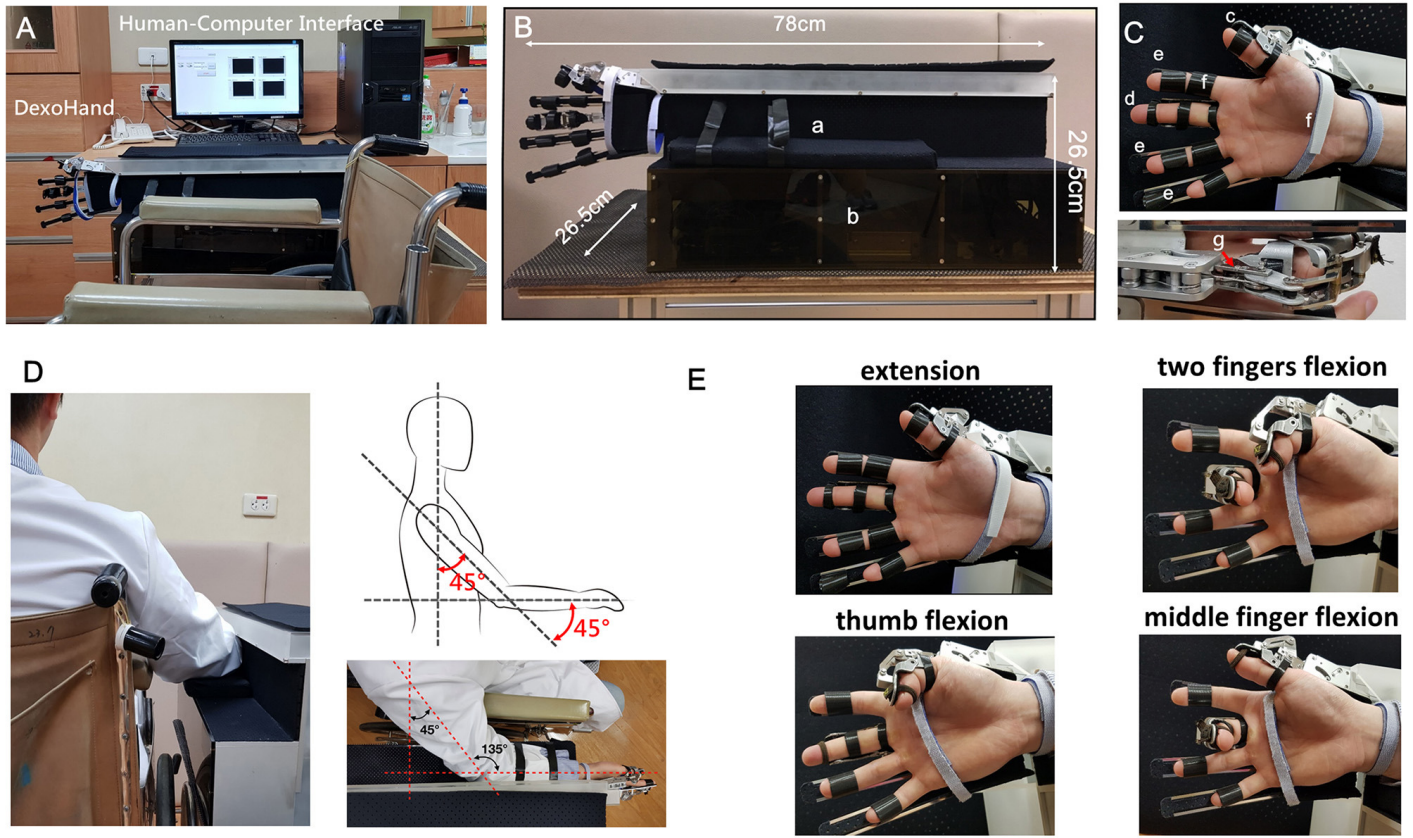
The application of *DexoHand* on a subject. **(A)** *DexoHand* and its computer-human interface. **(B)** The dimensions of *DexoHand*. a: the forearm holder, b: the control box containing motors and the control system. **(C)** The exoskeleton parts. c: the thumb module; d: the middle finger module; e: the three finger modules for the index, ring, and little fingers; f: an example of a Velcro strap that secures the body segments; g: the cable that drives the movement of the finger module. **(D)** The experimental setup showing the upper limb positioning. **(E)** The finger postures manipulated by the exoskeleton for the thumb and the middle finger.

**Figure 2:**
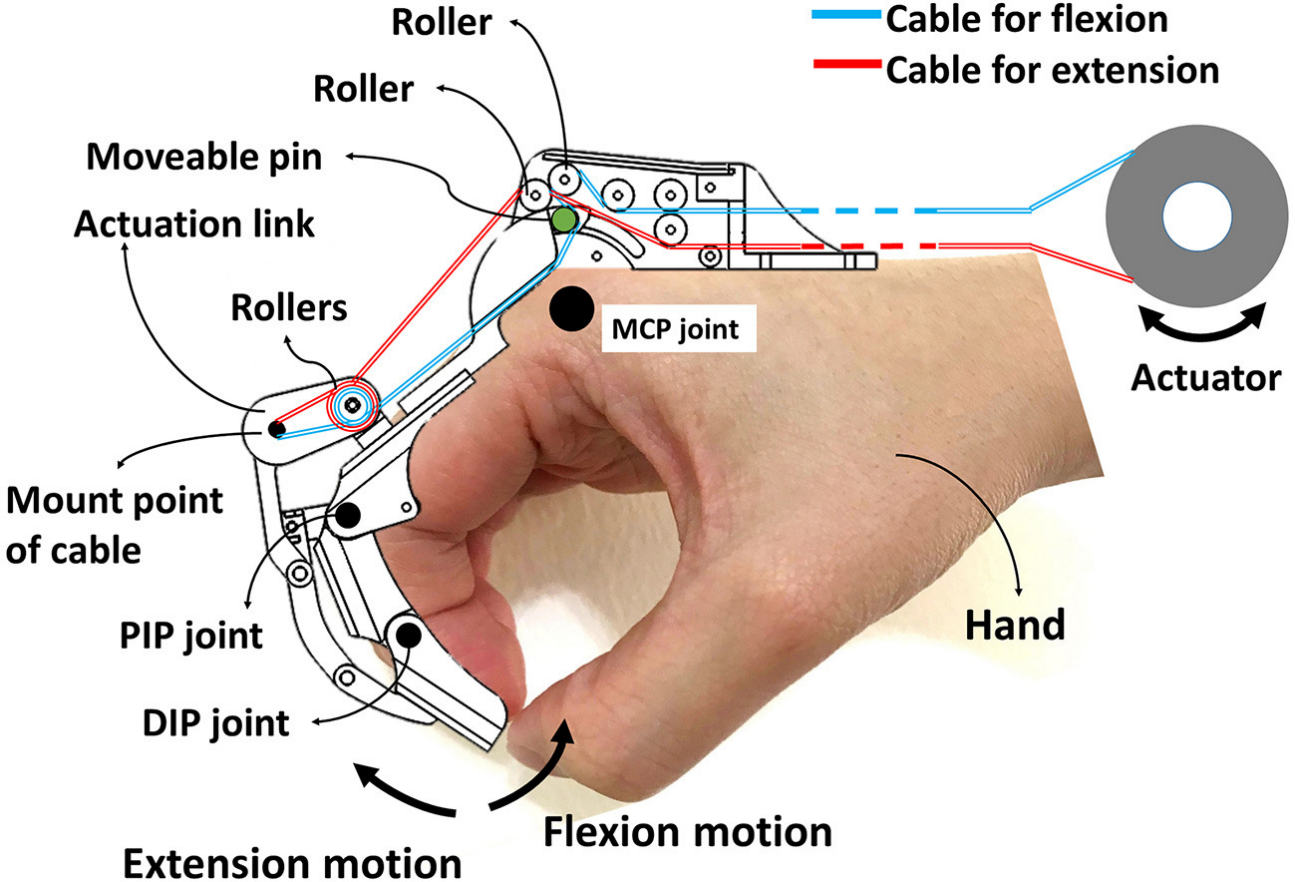
Cable routing layout for the *DexoHand*. The red and cyan lines represent cables used to drive extension and flexion motions, respectively. MCP joint movement is guided by the movable low-friction pin represented by the green circle. Four-bar linkage systems construct the mechanisms of the PIP, DIP, and IP joints of both finger models.

*DexoHand* and the human-computer interface are shown in Figure 1. The robot consists of a control box and two robotic fingers for the right middle finger and thumb (Figures 1A,B). These two fingers were specifically chosen because the middle finger has MCP, PIP, and DIP joints which is similar to the index, ring, and pinky fingers, and the thumb only has MCP and interphalangeal (IP) joints. The cables were driven by motors and the cables drove the patient's finger movement through changing the configuration of the exoskeleton (Figure 1C). During the development phase of the device, we defined the specifications as: (1) the device should have virtual centers of rotation that match that patient's MCP, PIP, DIP, and IP joints; (2) The force should be adequate to manipulating patients with spasticity, up to MAS = 3; (3) Finally, the patient should feel comfortable during the rehabilitation processes. To this end, the mechanical designs adopt a mechanism that has a virtual center of rotation that perfectly match that patient's finger joints by using the design from SW Pu et al. for the MCP joint and linkages for the PIP, DIP, and IP joints shown in the Figure 2. As the exoskeleton finger rotates, the center of the curved slot on the MCP joint is aligned with the rotation center of the hand's MCP joint. As shown in Figure 2, the red and cyan lines represent cables used to drive extension and flexion motions, respectively. The MCP joint movement is guided by the movable low-friction pin represented by the green circle in Figure 2, which moves along the curved slot that forms the desired movement trajectory. Several rollers that form a pulley system are mounted to reduce friction and adjust the transmission direction of the cable, enabling MCP flexion, and extension. The movement of PIP, DIP, and IP joints is coupled by a four-bar linkage system, which transmits angular motions and renders one-to-one correspondence for joint angles and the angular position of the actuator.

The actuators were chosen to have total estimated mechanical load of 98 N which was estimated adequate by computer simulations. The contact surfaces between the skin and the exoskeleton were placed with adequate padding so that comfort and safety are ensured. In addition, we found that forearm support is important to maintain a good posture of the proximal upper limb so that we installed a forearm support.

### Step 2: Subject Evaluation and Application of the *DexoHand*

The subjects were seated in a wheelchair for ROM and spasticity assessments and then received the CPM-based rehabilitation using *DexoHand*. The 22 subjects (4 healthy subjects and 18 stroke patients) received a follow-up evaluation 1 week after the first assessment to examine the test-retest reliability. Standard ROM assessment was performed on the MCP, PIP, and DIP joints of the right middle finger and the MCP and IP joints of the right thumb. Spasticity of the aforementioned joints was assessed using the MAS. The measurements were performed by a physiatrist.

After the ROM and spasticity assessments, *DexoHand* was placed on the subject's right hand with the shoulder at 45° flexion, elbow at 45° flexion, and forearm at 90° pronation position (palmar side inwards) (Figures 1D,E). Next, we first placed the middle finger and then the thumb on their corresponding finger parts of the *DexoHand*. The other three fingers, the index, ring, and pinky fingers were fixated by Velcro straps on simple finger holders that hold the fingers in neutral positions with the MCP, PIP, and DIP fingers in 0°. Finally, the wrist and forearm were secured on the forearm holder. The subject received a CPM rehabilitation protocol, which included the single-finger and two-finger conditions:

- The one-finger condition: The thumb or the middle finger was moved passively with the angular velocities of 15°/s or 150°/s. The sequence was thumb 15°/s, middle finger 15°/s, thumb 150°/s, and middle finger 150°/s, and each of the finger-velocity block had 10 repetitions. There was a 1-min rest between blocks.
- The two-finger condition: The thumb and the middle finger were simultaneously moved at an equal angular velocity of 90°/s with 200 repetitions.

One-finger condition was first performed followed by the two-finger condition. The CPM moved the thumb and the middle finger to-and-fro between 0° (neutral) to 90° flexion and 0° (neutral) to 150° flexion, respectively. The aforementioned angles are the sum of MCP, PIP, and DIP joint angles for the middle finger and the sum of MCP and IP joint angles for the thumb. During the experiment, the patient was asked to report pain any time when pain was perceived. As stated in the informed consent, we will immediately stop the experiment if the patient reported pain.

During the CPM, the cable tension of the flexion and extension cables was measured by force gauges (MT-25, Esense Scientific Ltd, Taiwan) and recorded through a signal acquisition card with a sampling rate of 200 Hz (NI Myrio-1900, The National Instruments Corp., USA) on the computer. The raw tension force was monitored by the sensor output voltage and then transferred to the cable tension by Equations (1, 2). Finally, the tension force alteration was computed by Equation (1). We used its highest peak (adjusted by its lowest peak) to represent the maximal force used when manipulating the finger:

(1) $$\begin{array}{l} \text{Resultant Force }\left(\text{RF}\right)\;=\text{sensor output voltage}/0.0212 \end{array}$$

(2) $$\begin{array}{l} \text{Cable Tension }\left(\text{CT}\right)\;=\;\left(\text{RF}/0.0212\right)/\left(2\times \text{cos}\left(\varepsilon \right)\right) \end{array}$$

(3) $$\begin{array}{l} \text{Force Alteration }\left(\text{FA}\right)\;=\;|\left(\text{C}{\text{T}}_{\text{highest peak}}\;-\text{C}{\text{T}}_{\text{baseline}}\right)| \end{array}$$

**Figure 3:**
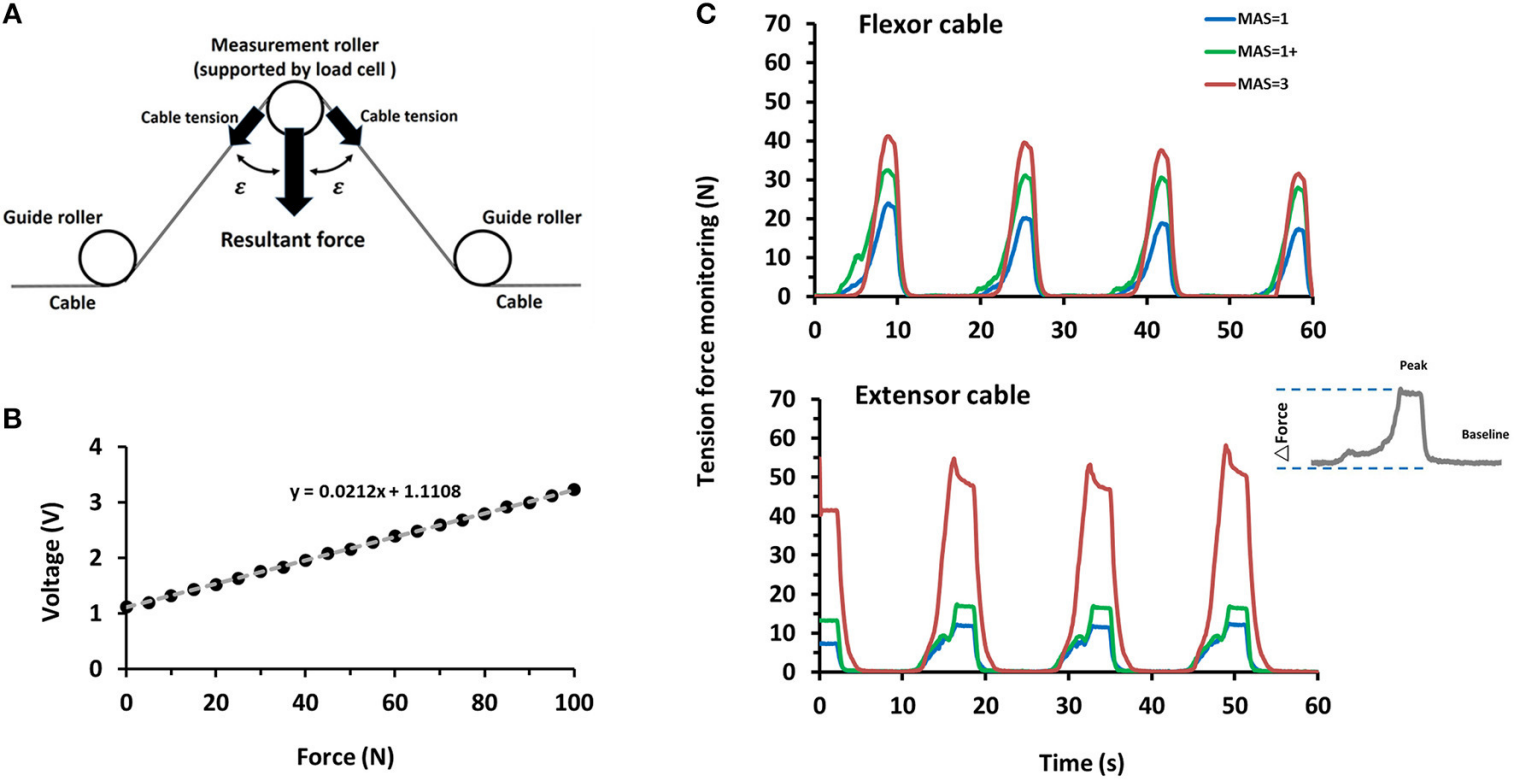
Example of cable tension profiles measured by the sensors imbedded in *DexoHand*. **(A)** The cable tension force was computed by taking the resultant force of the strain gauge, ε = 72.5°. **(B)** The calibration profile was used to infer the tension force from the voltage recorded by the strain gauge, *R*^2^ = 0.9997. **(C)** The cable tension profile was obtained through the cable tension monitor as the middle finger module performed a repetitive flexion-extension movement. Velocity = 15°/s. The upper and lower panels are the data obtained from the flexor and extensor cables, respectively. The data were obtained from the sample patients with the MAS = 1, 1+, and 3. The inset on the right denotes the data collected from a movement cycle example through the extensor cable, which was further used to compute the force change (ΔForce).

Where ε = 72.5° (Figure 3A), the constant of 0.0212 was obtained by the force calibration testing using the standard force ranged from 0 to 100 N on the tension sensor (*R*^2^ = 0.9997, Figure 3B).

### Step 3: Usability and Satisfaction Assessments

The usability assessment focused on the actual use of *DexoHand* and divided subjects into four groups: healthy (*n* = 4), MAS _≦1_ (*n* = 9), MAS_1+_ (*n* = 5), and MAS _≧2_ (*n* = 4). After applying *DexoHand*, the subjects completed the System Usability Scale (SUS) questionnaire. The questionnaire collected subjective evaluations and recommendations regarding the device. The SUS was developed by Brooke (1996) as a system usability tool, which has been widely used in the evaluation of a range of systems (Brooke, 2013). It is composed of 10 statements, each of which has a five-point scale that ranges from “Strongly Disagree” to “Strongly Agree.” There are five positive statements and five negative statements, which are presented in alternation. Odd numbered questions, Q1, 3, 5, 7, and 9 were positive questions, and the recorded scores were the original scores subtracted by 1 (Equation 4):

(4) $$\begin{array}{l} \text{Recorded scores }=\text{Original scores}-1 \end{array}$$

Even numbered questions, Q2, 4, 6, 8, and 10 were negative questions, and the recorded scores were 5 minus the original scores (Equation 5):

(5) $$\begin{array}{l} \text{Recorded scores }=\;5-\text{Original scores} \end{array}$$

The recorded scores from the two questions were summed up and then multiplied by 2.5 to yield a total SUS score that ranged from 0 to 100 (Equation 6):

(6) $$\begin{array}{l} \text{The total SUS score }=\text{Sum of the recorded scores }\times \;2.5 \end{array}$$

For the satisfication assessment, the responses to each of the five items in the satisfication questionnaire was rated on a 5-point Likert-type scale (5: very good; 4: good; 3: average; 2: poor; 1: very poor; Likert, 1932). The five items were averaged to yield the composite satisfaction scale ranging from 1 (least satisfactory) to 5 (highest satisfactory).

### Statistics and Reliability Analysis

To test the split-half reliability for the SUS, the correlation coefficent (r) between the scores obtained from the odd and even numbered SUS questions was first computed and the Spearman-Brown correction (ρ) was used to yield the predicted reliability value (ρ) (Equation 7):

(7) $$\begin{array}{l} \rho =\frac{2\times r}{1+r} \end{array}$$

The construct reliability of the satisfaction questionnaire that yields the composite satisfaction scale was assessed using Cronbach's α. The test-retest reliability for ROM, the MAS and cable tension force were performed using intraclass correlation coefficient (ICC). An ICC value between 0.75 and 1.00 was considered excellent, between 0.60 to 0.75 was good, between 0.40 to 0.59 was fair, and <0.40 was poor (Cicchetti, 1994).

The percent difference was used reflect the precision of the tension force under no-load condition and define the value of percentage difference <2.5% as negligible. The formula is as follows (Equation 8):

(8) $$\begin{array}{l} \text{Percent difference}=\frac{\left|f2-f1\right|}{\left(f2+f1\right)/2}\;\times \;100\% \end{array}$$

f2 and f1 denote the first and second measures for a measurement pair. The for each measurement pair was averaged to yeild the mean percent difference that reflects the precision.

Comparisons of the results across the three patient groups were performed using one-way ANOVA. Comparison between items, such as those of the SUS and satisfaction questionnaires, were compared using repeated-measures ANOVA. All data were presented as mean ± SD. Significance level was set as *p* < 0.05.

## Results

### Needs Definition

Based on the characteristics of patients with upper motor neuron syndromes and rehabilitation for neurological disorders, we delineated the following clinical situations of a hand rehabilitation robot: (1) the patients may have difficulty wearing the device due to spasticity, impaired motor control, and weakness; (2) a rehabilitation movement may need to be repeated up to 1,000 times for a training session; (3) each patient's velocity of training movement needs to be adjustable to avoid injury; (4) the forces that manipulate the hand should be monitored to avoid high force condition; (5) automatic methods should be applied for rehabilitation and risk management to reduce the manpower of monitoring patients.

### The Design Concept of *DexoHand*

Based on the aforementioned design specifications and the engineering concept, we developed the prototype *DexoHand*. The device was controlled by a human-computer interface through which the motion velocity and angle can be adjusted. The exoskeleton of *DexoHand* consisted of the thumb and middle finger modules and simple finger supports for the remaining three fingers. Each of the finger modules was driven by the flexor and extensor cables. The cables were driven by a servo motor (BLDC-Motor, force = 0.014 N-m, Faulhaber GmbH & Co. KG, Schönaich, Germany) coupled with a decelerator (gear ratio = 1:100). Through the decelerator, the torque output could reach up to 1.4 N-m, yielding a corresponding flexion and extension force of 145 and 112 N. According to our estimation, this force level is adequate to manipulate the fingers with MAS ≦ 3. Each flexor and extensor cable was installed with a tension sensor to monitor the cable tension profile during rehabilitation.

### Assessments

#### Reliability of MAS and ROM Assessments

Twenty-two subjects participated in this experiment and none of them reported previous experience in robotic rehabilitation. The ICC for MAS measurements for the first and second measurements was 1.00. The ICCs were 1.00 and 0.99 for the ROM of MCP and DIP joints of the thumb, and 0.97, 0.96, and 1.00 for the ROM of MCP, PIP, and DIP of the middle finger, respectively. The excellent test-retest reliability supports our approach of assigning patients into three muscle tone groups (MAS _≦1_, MAS_1+_, and MAS _≧2_).

#### Usability Assessment

Table 2 shows the scores for each SUS item in the healthy and patient subjects. We first calculated the total SUS score from the questionnaires obtained from all the subjects. Most of the items had high scores (all ≧ 3.75) except for item Q8, “I require technical assistance to use the exoskeletal robotic device,” which scored only 2.50 ± 1.29 in healthy subjects and 2.00 ± 0.77 in patient subjects. The overall usability scale among patient subjects revealed fair construct reliability of SUS (Cronbach's α = 0.64) and a high acceptance (94.58 ± 2.88, *n* = 18), indicating an excellent level of usability.

**Table 2:** SUS score obtained from the subjects.

**Item**	**Content**	**Score^a^**				
		**Healthy subject (*n* = 4)**	**All Patients (*n* = 18)**	**MAS _**≦1**_ (*n* = 9)**	**MAS_**1+**_ (*n* = 5)**	**MAS _**≧2**_ (*n* = 4)**
Q1	I would like to use the exoskeletal robotic device often	3.75 ± 0.50	4.00 ± 0.00	4.00 ± 0.00	4.00 ± 0.00	4.00 ± 0.00
Q2	I think the exoskeletal robotic device is complex to use	4.00 ± 0.00	3.94 ± 0.24	3.89 ± 0.31	4.00 ± 0.00	4.00 ± 0.00
Q3	I think the exoskeletal robotic device is easy to use	4.00 ± 0.00	4.00 ± 0.00	4.00 ± 0.00	4.00 ± 0.00	4.00 ± 0.00
Q4	I required technical assistance to use the exoskeletal robotic device	2.50 ± 1.29	2.00 ± 0.77	1.89 ± 0.57	2.60 ± 0.49	1.50 ± 0.87
Q5	I think the functionalities of the exoskeletal robotic device are well integrated	4.00 ± 0.00	4.00 ± 0.00	4.00 ± 0.00	4.00 ± 0.00	4.00 ± 0.00
Q6	I think the functionalities of the exoskeletal robotic device are not consistent	4.00 ± 0.00	4.00 ± 0.00	4.00 ± 0.00	4.00 ± 0.00	4.00 ± 0.00
Q7	I think most users can quickly learn to use the exoskeletal robotic device	4.00 ± 0.00	4.00 ± 0.00	4.00 ± 0.00	4.00 ± 0.00	4.00 ± 0.00
Q8	I think most users have difficulties learning to use the exoskeletal robotic device	4.00 ± 0.00	4.00 ± 0.00	4.00 ± 0.00	4.00 ± 0.00	4.00 ± 0.00
Q9	I am confident when using the exoskeletal robotic device	4.00 ± 0.00	3.88 ± 0.47	4.00 ± 0.00	4.00 ± 0.00	3.50 ± 0.87
Q10	I need to learn more background information of the exoskeletal robotic device before use	4.00 ± 0.00	4.00 ± 0.00	4.00 ± 0.00	4.00 ± 0.00	4.00 ± 0.00

a *Positive question SUS Score, original SUS Score-1; Negative question SUS Score, 5-original SUS Score*.

**Figure 4:**
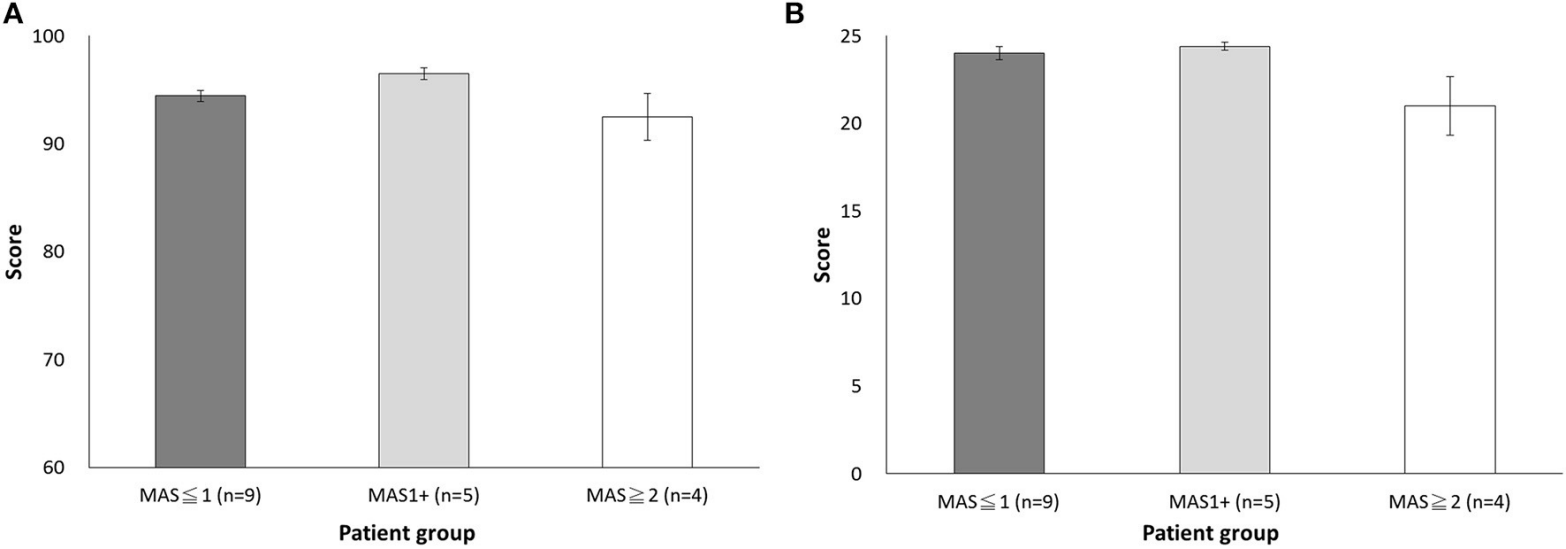
Comparison of the scores among the three muscle tone groups (MAS _≦1_, MAS_1+_, and MAS _≧2_) **(A)** Total SUS score. **(B)** Total satisfaction score.

The next question is whether usability is affected by the subjects' muscle tone. The results showed that the total SUS scores did not differ in MAS _≦1_ (94.44 ± 1.57), MAS_1+_ (96.50 ± 1.37), and MAS _≧2_ (92.5 ± 5.00) groups (*p* = 0.19, one-way ANOVA) (Figure 4A).

We further examined the characteristics of the Q4 item score among different spasticity level in the patient subjects to see if the high spasticity level group needed more assistance when wearing the device. The comparison among the three muscle tone groups showed that the score of Q4 item did not differ among the MAS _≦1_ (1.89 ± 0.36), MAS_1+_ (2.60 ± 0.30), and MAS _≧2_ (1.50 ± 1.00) groups (*p* = 0.076), but the Q4 item score in all patient subjects was low and hinted that the patient needed assistance when wearing the device. Therefore, the difficulty of wearing the device could substantially affect the usability, which is a problem universally observed in all patients regardless of their muscle tone levels.

To ensure that the patients were confident in the device, we analyzed the score of item Q9 “I am confident when using the exoskeletal robotic device.” We found comparable scores in the MAS _≦1_ (1.89 ± 0.36), MAS_1+_ (2.60 ± 0.30), and MAS (1.50 ± 1.00) groups. However, one case with the MAS = 3 in the MAS _≧2_ group showed low acceptance with the lowest item score of 2, indicating that patients with high levels of spasticity may consider the use of rehabilitation robot risky. This psychological factor accompanying the neurological status may affect the adoption of robot-assisted rehabilitation.

#### Satisfaction Assessment

The Cronbach's α of satisfaction assessment was 0.85, indicating good construct reliability. We then analyzed the difference in scores across items and found that the item of “Easy Wearability Satisfaction” had the lowest item scores (*P* < 0.05), a finding that is reminiscent of the results in SUS item Q4 (Table 3). Furthermore, the score was significantly different across muscle tone groups. Specifically, the MAS _≧2_ group had lower scores of 3.25 ± 0.92 than that of the MAS _≦1_ group (4.22 ± 0.44) and the MAS_1+_ group (4.40 ± 0.30). However, no statistical significance was found (*p* = 0.059), suggesting patients may need assistance from others (Figure 4B). Overall, all patient subjects revealed high satisfaction with *DexoHand* (23.44 ± 2.28). The results showed that patient subjects did not report any pain during the experiment. The satisfaction questionnaire showed nearly highest satisfactory (4.83 ± 0.38) in the item of “Comfort Satisfaction” in the patient group, again supporting that minimal or no discomfort was perceived during the whole course of experiment.

**Table 3:** Satisfaction score in healthy subjects and muscle tone groups.

**Item**	**Question**	**Group**				
		**Healthy subject (*n* = 4)**	**All Patients (*n* = 18)**	**MAS _**≦1**_ (*n* = 9)**	**MAS_**1+**_ (*n* = 5)**	**MAS _**≧2**_ (*n* = 4)**
Overall satisfaction	How do you rate *DexoHand* ?^†^	5.00 ± 0.00	4.89 ± 0.32	5.00 ± 0.00	5.00 ± 0.00	4.50 ± 0.58
Comfort	How do you rate the comfort of *DexoHand* ?	5.00 ± 0.00	4.83 ± 0.38	4.89 ± 0.33	5.00 ± 0.00	4.50 ± 0.58
Easy wearability	How do you rate the easy wearability of *DexoHand* ?	4.75 ± 0.50	4.06 ± 0.80	4.22 ± 0.67	4.40 ± 0.55	3.25 ± 0.96
Joint movement	How do you rate the joint movement of *DexoHand* ?	5.00 ± 0.00	4.83 ± 0.71	5.00 ± 0.00	5.00 ± 0.00	4.25 ± 1.50
Rehabilitation use	How did DexoHand help with your rehabilitation ?	5.00 ± 0.00	4.83 ± 0.38	4.89 ± 3.33	5.00 ± 0.00	4.50 ± 0.58

† *The rating was defined from a scale of 1 to 5*.

### Cable Tension Monitoring

**Figure 5:**
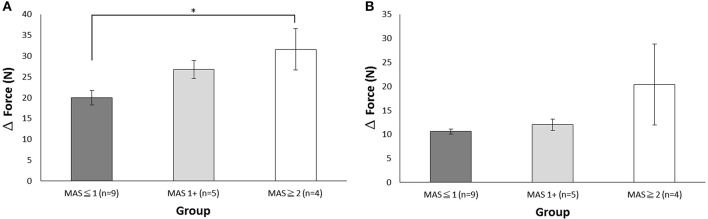
The maximum delta force during the flexion and extension movements of the middle finger module. **(A)** The maximum delta force of the flexor cable during flexion. **(B)** The maximum delta force of the extensor cable during extension. The data is presented with mean and standard error of mean. *p < 0.05.

To investigate the concept of integration of sensor technology and robot, we integrated a tension sensor into the flexor and extensor cables in each finger module to monitor the tension force profile of the subjects' fingers during *DexoHand* manipulation. Figure 3 showed the tension force profile obtained from four example subjects in the Healthy, MAS _≦_1, MAS1+, and MAS _≧_2 groups. As the finger module performs alternating flexion and extension movements, the tension correspondingly increased in the flexor (Figure 3C, upper panel) and extensor (Figure 3C, lower panel) cables. In the middle finger module, patients in the MAS _≧2_ and MAS _≦1_ groups had the highest and lowest maximum delta force, respectively, during finger flexion (Figure 5A) [*F*_(2, 15)_ = 3.995, *p* = 0.041]. However, this difference was not found during finger extension [*F*_(2, 15)_ = 1.712, *p* = 0.214] (Figure 5B).

Finally, we examined the test-retest reliability of the tension force under no-load condition, during which no human fingers were applied. The results showed that the percentage differences were 1.34% for flexion and 0.41% for extension in the middle finger module, and were 1.27% for flexion and 0.66% for extension in the thumb module, a finding indicating that the precision of the sensor system was within the target level.

## Discussion

Given the novelty of rehabilitation robots, more, and more pioneering designs are being developed for clinical applications of rehabilitation for neurological disorders. The significance of this study is to develop a wearable exoskeletal robotic device through a design process emphasizing user experience, utility, and safety-related tension profiles to ensure that the clinical needs are met. Furthermore, healthy subjects and stroke patients with different levels of spasticity were recruited to examine different responses across subject subtypes.

After examining the usability of *DexoHand* in a real rehabilitation environment, the SUS usability questionnaire showed good internal consistency except item Q4. The satisfaction results showed positive feedback, indicating that patients are willing to use the device for rehabilitation. Finally, the perceived usability of the total SUS scores did not differ across subject groups, indicating that all types of subjects, even the stroke patients with spasticity who were considered to have soft tissue strain during therapy, gave positive commendation.

Item Q4 in the SUS had the lowest score and this property was universally observed in all subject groups including the control group. This finding suggests that wearing an exoskeletal robot is inevitably time consuming and may require the help of others. The same problem that patients who manifested spasticity in fingers, hands and forearms had difficulty wearing hand exoskeletons by themselves also was observed in previous study (Almenara et al., 2017). It is one of the major problems in the design of rehabilitation exoskeleton robots and will be the major line of inquiry in future. Any breakthrough in this field is expected to improve patient satisfaction and clinical usability, thereby greatly increasing the adoption rate of these devices. For the first item of the SUS questionnaire, “I would like to use the exoskeletal robotic device often” would not be completely suitable for healthy subjects. Specifically, a healthy subject does not need rehabilitation so that the rating is lower as compared to other items. However, this question is required for a SUS questionnaire according to the original design of SUS (Brooke, 2013). Accordingly, the validity of Item 1 in the healthy subject needs to be carefully interpreted.

Form the engineering point of view, direct-drive robots are relatively easy to control, so most rehabilitation robots are applied with direct-drive design. However, this study used a different concept by driving the robot using cables while simultaneously measuring cable tension. The combination of mechanical design and measurement of load cell signals offers a unique opportunity to monitor the interaction between the subject and the robot.

Patients with spasticity usually manifest with a dynamic increase in muscle tone when the limb briskly stretched by an external force. Robot-assisted rehabilitation is based in part on the concept of manipulating limbs in a continuous and repeatable manner (Heo et al., 2012; Lum et al., 2012). However, the safety of patients with severe spasticity is not guaranteed when applying the device automatically with minimal personal supervision. Therefore, risk management has become the major issue. To this end, we use cable tension sensor in *DexoHand* as an attempt to develop a risk management strategy. The results of this study revealed perfect reliability under zero-load condition and showed minor differences between healthy subjects and patients. The intended use of cable tension was risk management. When a patient is in need of excessive force to manipulate a limb (which is expected to cause discomfort or injury), we can observe the maximum value. Almost all subjects in this study had the MAS ≦ 3 and thus their fingers can be manipulated relatively easily by *DexoHand*. Future studies will apply *DexoHand* in other patient groups to examine its value for risk management.

The subjects recruited in this study had more male and less female, a property that was determined by the size of the exoskeleton hand we used. Indeed, different genders can have different experiences, such as for pain and satisfaction. Further studies might apply a verity of exoskeleton sizes to avoid this gender bias.

Different design concepts yield different scenarios for clinical applications. Specifically, when designing exoskeletal robots for rehabilitation, the trade-off between the use of rigid robots, such as those using rigid bodies and joints, and soft robots, such as those using fabrics or other flexible materials, is important in determine a plausible mechanical design. Considering the spastic condition in patients with upper motor neuron syndrome, we chose to design an exoskeleton mainly made by aluminum. However, patients wearing the device reported fitting problems such that substantial efforts are needed to make sure the centers of rotation match between the robot and the human finger joints (Yang et al., 2004). In addition, this design also made it too heavy for patients with weak muscle strength in their shoulder and arm to use as a functional aid in daily living. Therefore, future works on a light weight design are needed (Alami et al., 2006).

## Conclusions

The subjects, including the healthy subjects and patients with different levels of spasticity, received *DexoHand* therapy and reported excellent usability and satisfaction. The integration between mechanical design and cable tension sensors made it possible for us to monitor the interaction between human and robot during therapy. In future studies, we will explore risk management using this design and apply neurorehabilitation to patients with upper motor neuron disease.

## Data Availability Statement

Data and materials can be made available upon request to the authors.

## Author Contributions

Y-LT, J-JH, S-WP, H-PC, S-CH, J-YC, and Y-CP designed and conducted the experiments. Y-LT, J-JH, and H-PC analyzed and interpreted the data. Y-LT, J-JH, S-WP, H-PC, J-YC, and Y-CP wrote the manuscript. All authors provided critical feedback on the manuscript. All authors read and approved the final manuscript.

### Conflict of Interest Statement

The authors declare that the research was conducted in the absence of any commercial or financial relationships that could be construed as a potential conflict of interest.

## Abbreviations

CPM: continuous passive movement
MAS: Modified Ashworth scale
ROM: range of motion
SUS: System usability scale
MCP: middle carpal phalangeal
PIP: proximal interphalangeal
DIP: distal interphalangeal
IP: interphalangeal
RF: resultant force
CT: cable tension
FA: tension force alteration
ICC: intraclass correlation coefficient.
